# Diagnosis of Joubert Syndrome 10 in a Fetus with Suspected Dandy-Walker Variant by WES: A Novel Splicing Mutation in* OFD1*

**DOI:** 10.1155/2018/4032543

**Published:** 2018-11-15

**Authors:** Siyuan Linpeng, Jing Liu, Jianyan Pan, Yingxi Cao, Yanling Teng, Desheng Liang, Zhuo Li, Lingqian Wu

**Affiliations:** ^1^Center for Medical Genetics, School of Life Sciences, Central South University, Changsha, Hunan, China; ^2^Prenatal Diagnosis Center of Hunan Province, Hunan Provincial Maternal and Child Health Care Hospital, Changsha, Hunan, China; ^3^Hunan Jiahui Genetics Hospital, Changsha, Hunan, China

## Abstract

Joubert syndrome (JBTS) is a clinically and genetically heterogeneous group of ciliary diseases. To date, 34 subtypes of JBTS have been classified due to different causative genes or extra clinical features. Most of them are autosomal recessive, while only the subtype 10 (JBTS10) is a quite rare X-linked recessive disorder caused by* OFD1* mutations with few reports. In this study, by using whole exome sequencing (WES), a novel* OFD1* splicing mutation (c.2488+2T>C) was identified in a male fetus with suspected Dandy-Walker variant (DWV) and syndactyly, for whom abnormal karyotype and pathogenic CNV have been excluded. This mutation was inherited from the mother who has experienced two similar pregnancies before. An abnormal skipping of exon 18 in* OFD1* mRNA was confirmed by RT-PCR and sequencing. Result from quantitative RT-PCR also showed that total* OFD1* mRNA in the index fetus was significantly lower than the control. After a combined analysis of genetic testing results and genotype-phenotype correlations, the novel mutation c.2488+2T>C in* OFD1* was considered to be the genetic cause for the affected fetus. Thus the diagnosis should be JBTS10 rather than the primary clinical diagnosis of DWV. We report the first prenatal case of JBTS10 in Chinese population, which not only helps the family to predict recurrence risks for future pregnancies but also provides more information for understanding such a rare disease. The results also present evidence that WES is an effective method in prenatal diagnosis for those fetuses with Joubert syndrome.

## 1. Introduction

Joubert syndrome (JBTS) is characterized by cerebellar vermian, mid-hindbrain dysgenesis, cerebellar ataxia, developmental delay, oculomotor apraxia, and abnormalities in breathing patterns [1]. The unique cerebellar and brainstem malformation called “molar tooth sign” (MTS) on brain magnetic resonance imaging (MRI) is a characteristic neuroradiological hallmark of typical JBTS [2]. In addition, retinal dystrophy, cystic kidneys, and polydactyly are also seen in some patients [3].

JBTS is a clinically and genetically heterogeneous group of ciliary diseases. To date, 34 subtypes of JBTS have been classified due to different causative genes or extra clinical features. Most of JBTS are autosomal recessive, while only the subtype 10 (JBTS10) is X-linked recessive disorder caused by* OFD1* mutations. The variants of* OFD1* have been known to cause several genetic disorders. The most common is orofaciodigital syndrome I (OFD1 syndrome, MIM 311200), which is a male-lethal X-linked dominant disease [4]. Three other rare recessive X-linked disorders include Joubert syndrome 10 (JBTS10, MIM 300804) [5–7], Simpson-Golabi-Behmel syndrome, type 2 (SGBS2, MIM 300209) [8], and a severe retinitis pigmentosa (RP32, MIM 300424) [9]. Since the first JBTS10 case was identified and reported in 2009, only less than 20 families with male patients have been reported to be JBTS10 [5]. So it is still hard to gain a comprehensive understanding for such a very rare disease.

In a previous study, approximately 10% of patients with JBTS who had abnormal cerebrospinal fluid collections were misdiagnosed as DWV [10, 11]. Generally speaking, the majority of patients with JBTS show global developmental delay, while a certain proportion of patients with DWV usually possess normal cognitive functions [10, 12]. This indicates that the prognosis between JBTS and DWV is greatly different. Therefore, a definitive diagnosis between JBTS and DWV is critical for patient's management, disease prediction, and genetic counseling. Quantitative assessment of brainstem development has strongly suggested that brainstem anatomy, specifically isthmus dimensions, can be used to radiologically distinguish between Dandy-Walker syndrome and Joubert syndrome in children with hypotonia, ataxia, and developmental delay [10]. Thus the molar tooth sign on axial imaging through the brainstem isthmus can effectively distinguish between Joubert and Dandy-Walker syndromes [10]. However, for most of prenatal cases, the abnormalities detected by prenatal ultrasound screening in fetuses at high-risk of Joubert syndrome are usually nonspecific, like cerebellar vermis aplasia/hypoplasia, ventriculomegaly and so on. What is more, the cerebellar vermis is a relatively late-developing structure and may not cover the fourth ventricle until 18 weeks' gestation, making visualization of the molar tooth sign (MTS) difficult earlier in gestation [13]. In order to accurately diagnose JBTS in prenatal diagnosis, it is recommended that serial ultrasounds starting at 11 weeks and followed by fetal MRI at 20-22 weeks of gestation [14]. But sometimes it is difficult to implement in clinical practice in China. Because when the fetus displays the suspected DWV at first or second trimester of pregnancy, some parents may worry about that the fetus would suffer from severe symptoms and choose artificial abortion, even after well-informed and genetic counseling. This will result in the absence of further detection and miss more clinical information, such as “molar tooth sign” revealed by prenatal MRI. Molecular genetic analysis along with imaging findings may be a good choice to help the early prenatal diagnosis and uncover the potential genetic etiology.

In this study, we described a nonconsanguineous healthy Chinese couple with three fetuses, who were all suspected to suffer from DWV by prenatal ultrasound screening. Further genetic testing, such as karyotype analysis, microarray, and WES, was followed up to make a certain diagnosis to this family. The evaluation based on all the results and genotype-phenotype analysis indicated that a novel splicing variant in* OFD1* gene would be the key pathogenic factor, suggesting a diagnosis of JBTS10 for the affected fetus rather than DWV.

## 2. Materials and Methods

### 2.1. Clinical Features

The index fetus (II:4, Figure 1(a)) was the third child of a nonconsanguineous healthy Chinese couple with an unremarkable family history. Prenatal systematic ultrasound was performed at 22 weeks of gestation. Imaging manifestations included hypoplastic cerebellum and absent vermis with enlarged lateral ventricles. Bilateral postaxial polydactyly on hands was observed when her pregnancy was terminated by induction of labor at 25 weeks of gestation. No obvious malformation was observed on the appearance of the induced fetus. The parent refused to conduct further autopsy. The previous two fetuses (II:2 and II:3, Figure 1(a)) had been artificially aborted also due to their abnormal ultrasonic characteristics of brain similar to those of the index fetus, such as hypoplastic cerebellum and absent vermis, enlarged posterior fossa, and hydrocephalus in the second fetus and vermis hypoplasia in the first fetus. These characteristics on brain were suspected to be DWV. The three fetuses were all male observed after abortion. The first child (II:1, Figure 1(a)) of the mother with her unrelated healthy ex-husband was a boy, who was born at full term. He was unable to hold his head up and died of unknown causes at the age of 13 months. No further clinical data of him was available. The mother (I:2) is a 30-year-old healthy woman with neither observable facial abnormalities nor postaxial polydactyly. Her development history was normal. And she got her high school diploma at age 19 years while IQ test had never been performed. Magnetic Resonance Imaging (MRI) on her head was normal at age 30 years when the couple was referred to our clinic for genetics counseling.

### 2.2. Karyotype Analysis and Microarray Analysis

This study was approved by the Ethics Board of School of Life Sciences, Central South University. Informed consent was obtained from the patients of this family. G-banding analysis at a level of 550 bands was carried out using metaphases prepared by standard methods from umbilical cord blood of the index fetus. Genome-wide copy number variation (CNV) analysis of genomic DNA from the index fetus and his asymptomatic mother were performed by using Illumina HumanCyto-SNP12 chip (Illumina, San Diego, CA). Fluorescence in situ hybridization (FISH) analysis was performed to confirm the possible pathogenic CNVs by using BAC probe RP11-7N21 (red) within the deletion region of 8q21.2 and a control probe RP11-714N16 (green) mapped to 8q24.3 in the index fetus and the mother.

### 2.3. Whole Exome Sequencing

Whole exome sequencing (WES) was performed for the index fetus and the parents according to the manufacturer's protocols. The exomes were captured using the xGen® Exome Research Panel v1.0 (Integrated DNA Technologies) and sequenced on an Illumina Hiseq2000 (Illumina, San Diego, CA, USA) with 100-bp paired-end reads. Candidate pathogenic variants were confirmed by Sanger sequencing.

### 2.4. RNA Extraction and Reverse Transcription PCR

Total RNA was isolated from cultured lymphocyte cell line from the index fetus and healthy male control subject using TRIzol reagent (Invitrogen, Carlsbad, CA, USA) according to the manufacturer's protocol. One microgram of the extracted RNA was reverse transcribed into cDNA using RevertAid First Strand cDNA Synthesis Kit (Thermo scientific, Carlsbad, CA, USA). The effect of novel* OFD1* splicing variant on cDNA sequence was investigated using polyacrylamide gel electrophoresis and PCR-based Sanger sequencing. The primer sequences were 5′- CTTCCTCCAGACGCCTCTCTT-3′ (forward, in exon 16) and 5′- CGTTCCCTTTCTAAAACTTCTTGTA-3′ (reverse, in exon 20).

### 2.5. Real-Time Quantitative PCR

Real-time quantitative PCR (qPCR) was performed on Detection System (Roche, Boston, MA, USA) using SYBR Green qPCR Master Mix (Thermo Fisher, Carlsbad, CA, USA). The primer sequences were used with 5′-AGCCCAGTCTTTGGCAATAA-3′ (forward) and 5′-TTGTGCCAGAAGCTCCAGTT-3′ (reverse). The *β*-actin mRNA expression level was used as a reference. Three replicates were analyzed.

## 3. Results

### 3.1. Microarray Analysis and FISH Validation

**Figure 1:**
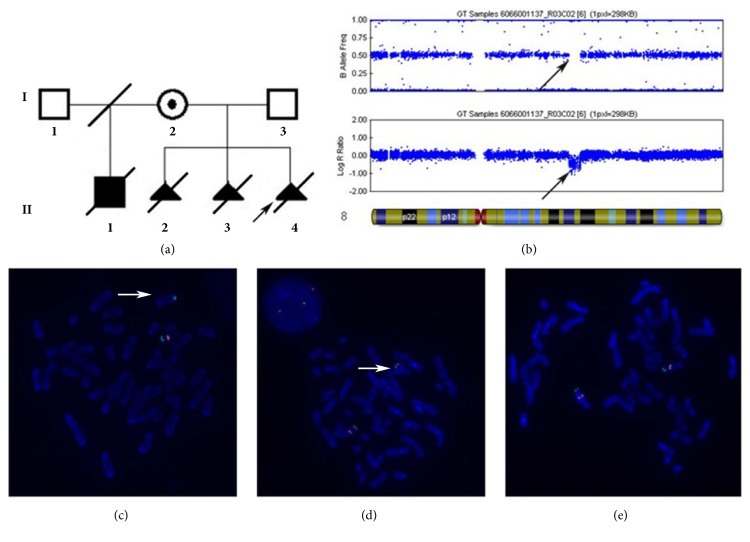
**Pedigree of the family, microarray analysis, and FISH results**. (a) Pedigree of the family. The arrow indicates the index fetus. Filled black symbols indicate the affected subjects. II:2, II:3, and II:4 were all male. (b) Plots of the copy number for individual SNP loci along chromosome 8 by Illumina HumanCytoSNP-12 BeadChip, showing a 4.9Mb (nt:82517136-87441476) heterozygous deletion 8q21.13-q21.3 in the fetus. Black arrow points to the deletion region. (c, d) FISH results of the index fetus and his mother. RP11-7N21 (red) mapped to the deletion region of 8q21.2 was used as the purpose probe, while RP11-714N16 (green) mapped to 8q24.3 as the control probe. Images showed no red signal onto the abnormal chromosome 8 in the metaphase cells of the index fetus (c) and the mother (d). (e) FISH result of the father. There are two signals of both red and green probes in the metaphase chromosome of the father (I:3).

G-banding analysis showed that the karyotype of the index fetus was 46, XY. Genome-wide copy number variation (CNV) analysis by using Illumina HumanCyto-SNP12 chip revealed a 4.9Mb heterozygous deletion at 8q21.13-q21.3 (nt: 82517136-87441476) in both the index fetus (Figure 1(b)) and the asymptomatic mother. Therefore, the karyotype was revised to be 46, XY, arr[hg19]8q21.13q21.3 (82517136-87441476) ×1. The deletion was confirmed by FISH using BAC probe RP11-7N21 (red) within the deletion region of 8q21.2 and a control probe RP11-714N16 (green) mapped to 8q24.3 in the index fetus and the parents. There was only one red signal onto the chromosome 8 in the metaphase cells of the index fetus and the mother, while the father had two red signals (Figures 1(c), 1(d), and 1(e)).

### 3.2. Identification of a Novel Hemizygous Variant in OFD1

**Figure 2:**
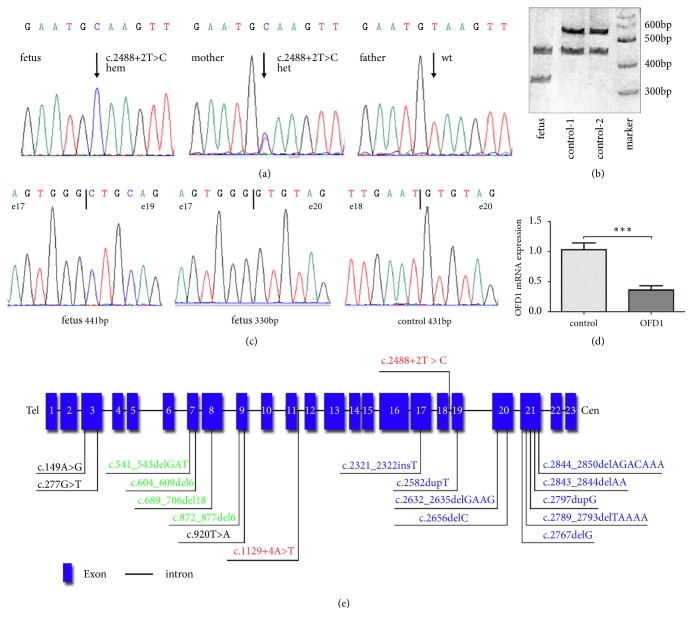
**Molecular findings from sequencing,* OFD1* expression assessment, and the distribution of mutations found to cause JBTS10**. (a) Sanger sequencing showed a hemizygous splice variant (c.2488+2T>C, NM_003611.2) in the* OFD1* gene in the index fetus, while heterozygous in the mother and absent in the father. (b) Polyacrylamide gel electrophoresis showed spanning exon amplification products in the index fetus and two male healthy controls. The controls displayed two bands, 431-bp and 541-bp, respectively. The index fetus had additional two bands, 441-bp and 330-bp, respectively. (c) Sanger sequencing showed that exon 19 was skipped in the 431-bp product from the healthy controls, which was confirmed to come from other normal transcript variants. In the affected fetus exon 18 was skipped in the 441-bp product, while exons 18 and 19 were both skipped in the 330-bp product (d) The qPCR result reveals the decrease of total* OFD1* mRNA in the index fetus. *∗∗∗* P < 0.001 (independent t -test). (e) Gene structure of* OFD1* and the distribution of mutations found to cause JBTS10. The blocks indicate the gene exons. All the mutations are collected from HGMD. The mutations marked with blue, green, black, and red correspond to frame-shift, in-frame, missense, and splicing mutations, respectively. Upper: the identified mutation in this study.

Whole exome sequencing identified a hemizygous variant of* OFD1* located on the short arm of chromosome X (c.2488+2T>C, NM_003611.2) in the index fetus. This variant located at the splice-donor site of intron 18 in* OFD1*, which was confirmed by Sanger sequencing, with heterozygous in the mother but absent in the father (Figure 2(a)). The variant has not been reported in all public databases and is predicted to destroy a normal splicing site by using the Human Splicing Finder (http://www.umd.be/HSF3/index.html).

### 3.3. Confirmation for a Skipping of Exon 18 and Total OFD1 mRNA Reduction

To evaluate the mutational effect of c.2488+2T>C, spanning exon amplification product was analyzed by polyacrylamide gel electrophoresis. It was strange that an additional amplification product (431-bp, wild-type allele is 541-bp) was observed in two healthy control subjects (Figure 2(b)). Direct sequencing verified that exon 19 was skipped in the 431-bp product. By querying this sequence in UCSC genome browser and BLAST in NCBI, the skipping of exon 19 matched the normal transcript variant X12 (XM_024452471.1) or transcript variant X6 (XM_011545597.2) in* OFD1*, which exists in blood but does not translate into protein. In the index fetus, two additional amplification products (441-bp and 330-bp) were observed. Direct sequencing verified a skipping of exon 18 in the 441-bp product, while a skipping of exons 18 and 19 exhibited in the 330-bp product (Figure 2(c)). These data indicated that c.2488+2T>C leads to a skipping of exon 18 in all transcript variants and is predicted to produce a premature stop codon (PTC) at amino acid position 840. Furthermore, RT-qPCR revealed a decrease of total* OFD1* mRNA in the affected fetus, which may be caused by nonsense-mediated decay (Figure 2(d)).

## 4. Discussion

Here we report a nonconsanguineous healthy Chinese couple with three male fetuses, all of whom were suspected to suffer from DWV by prenatal ultrasound screening. The index fetus also presented with bilateral postaxial polydactyly on hands. When structural abnormalities are detected by prenatal ultrasound screening in a fetus, chromosomal microarray analysis (CMA) revealed clinically relevant deletions or duplications in 6.0% fetuses with normal karyotype, so CMA should be recommended as the primary test [15, 16]. Hence, to uncover the potential genetic etiology for this family, we first performed microarray analysis in the index fetus and the mother, which revealed a 4.9Mb deletion of 8q21.13-q21.3 in both of them. This deletion has not been previously reported in the Database of Genomic Variants and the database of Decipher. Although the 4.9Mb deletion region encompasses more than 20 genes, none of them is related to DWV phenotypes or has been identified to be imprinted genes. In addition, the mother with the same deletion showed no abnormality. Therefore, we speculate that the 4.9Mb deletion may be not the cause for the affected fetus.

To further explore the pathogenic variants, we adopted the WES as the genetic testing for the index fetus. A novel hemizygous mutation in intron 18 of* OFD1* (c.2488+2T>C, NM_003611.2) was identified. To our knowledge, this mutation has not been reported in all public databases and is predicted to alter the normal splicing. Our following RT-PCR detection confirmed a skipping of exon 18 in the* OFD1* mRNA transcript due to the mutation, which would result a premature stop codon at amino acid position 840. Besides, total* OFD1* mRNA in the index fetus was also significantly lower than healthy male controls, which may be caused by nonsense-mediated decay. Taking all these findings into consideration, this novel mutation can be classified as “pathogenic” based on the ACMG guideline (Table S1) [17]. So the mutation c.2488+2T>C in* OFD1* was likely to be the genetic cause for the affected fetus.

According to the Human Gene Mutation Database (http://www.hgmd.cf.ac.uk/ac/index.php; update to April 2018), 157 natural inactivating mutations of* OFD1* gene have been identified in patients. OFD1 protein is located at the base of cilia and acts as an interacting partner of the LCA5-encoded ciliary protein lebercilin [5], which appears to play a critical role in the early development of many parts of the body, including the brain, face, limbs, and kidneys. The majority of the* OFD1 *mutations were associated with OFD1 syndrome, which is a male-lethal X-linked dominant disease characterized by malformations of the face, oral cavity, and digits. Other mutations have been reported to cause another three disorders, including JBTS10, SGBS2, and RP32.

Up to now, only 17 mutations of* OFD1* have been reported to cause JTBS10 (Figure 2(e)), including nine small deletion/insertion mutations, four in-frame deletions, three missense mutations, and a splicing mutation. Notably, all the nine frame-shift mutations located at the downstream of exon 17. The three missense mutations and four in-frame deletions which may not lead to OFD1 protein truncation and probably do not abolish its binding affinity to functionally interacting proteins, such as lebercilin [1, 18–23]. The only reported splicing mutation c.1129+4A>T located at intron 11 was considered to have broader clinical phenotype than JBTS, which may be defined to a newer classification as Joubert syndrome with oral-facial-digital defects [24].

The correlations between genotype and phenotype are beginning to be recognized with the expansion of the* OFD1* gene phenotype spectrum. Mutations located at the upstream of exon 16 almost are lethal for males and cause OFD1 syndrome in females, but truncated* OFD1* mutations at the downstream of exon 17 all lead to JBTS10. In this study, the novel variant c.2488+2T>C in the index fetus locates at downstream of exon 17 and causes a skipping of exon 18 in mRNA. Besides, the index male fetus did not have the typical facial features of OFD syndrome except for the postaxial polydactyly. Three male patients with similar phenotypes in this family were in line with the X-linked recessive genetic pattern. Together with the analysis for genotype-phenotype correlation and genetic pattern in the family, we speculate that the index fetus should suffer from JBTS10, but not OFD syndrome or the primary diagnosis of DWV. Unfortunately, all the three affected fetuses in this family were carried out artificial abortion at second trimester of pregnancy after the identification of suspected DWV, and no followed-up MRI was performed to verify whether they had a molar tooth sign on the brain. Thus, in prenatal condition, limited information from the fetus may cause clinical misdiagnosis, which could result in improper management for the pregnancy or counseling suggestion for their family.

With the development of genetic technologies, more and more advanced molecular genetic tests are adopted for prenatal diagnosis, such as CMA and WES. In 2018, ISPD, SMFM, and PQF released a joint position statement on genome-wide sequencing for fetal diagnosis, including “the use of diagnostic sequencing is currently being introduced for evaluation of fetuses for whom standard diagnostic genetic testing, such as CMA, has already been performed and is uninformative” [25]. Consistent with the statement, we first used G-banding and CMA to help the prenatal diagnosis for the affected family and finally identified the novel mutation of* OFD1* as the cause of disease via WES. Our case provides evidence that genome-wide sequencing, like WES, would be a helpful and effective testing in prenatal diagnosis, especially for those fetuses without definite clinical differential diagnosis. In addition, only one case of prenatal diagnosis for JBTS10 in Caucasian was reported since first JBTS10 was identified in 2009 [19]. Our study described the first prenatal case of JBTS10 in Chinese population, which not only helps the family to determine recurrence risks for future pregnancies due to the definite diagnosis but also provides more information and deeper understanding for such a rare disease.

To sum up, we identified a novel splicing variant of* OFD1* (c.2488+2T>C) for the first time via WES and determined it to be the genetic etiology for an affected family. According to the genetic testing results and genotype-phenotype analysis, a prenatal diagnosis of JBTS10 for the index fetus was determined, which corrected the primary clinical diagnosis of DWV. Our study suggested that WES would be a helpful testing to diagnose the pregnancies with multiple congenital anomalies detected by prenatal ultrasound, and an effective method for differential diagnosis between some rare diseases, such as JBTS and DWV, in early gestation.

## Data Availability

The data used to support the findings of this study are included within the article and the supplementary information file.

## Supplementary Materials

Supplementary Materials Table S1: evaluation of the pathogenicity of the novel variant c.2488+2T>C in* OFD1* gene.
