# Supplementary material for: Diagnosis of Joubert Syndrome 10 in a Fetus with Suspected Dandy-Walker Variant by WES: A Novel Splicing Mutation in OFD1
Source: Biomed Res Int. 2018 Nov 15;2018:4032543. doi: 10.1155/2018/4032543 (PMC6276521; doi:10.1155/2018/4032543)
Supplement: Supplementary Materials — Table S1: evaluation of the pathogenicity of the novel variant c.2488+2T>C in OFD1 gene. [file 4032543.f1.docx]

Table S1. Evaluation of the pathogenicity of the novel variant c.2488+2T>C in *OFD1* gene.

|  | *OFD1* c.2488+2T>C |
| --- | --- |
| Population data | Absent in population databases (PM2) |
| Computational and predictive data | Predicted null variant (+2 splice sites) in a gene where LOF is a known mechanism of disease (PVS1) and predicted to destroy normal splicing site by using the Human Splicing Finder (PP3) |
| Functional data | Destroy the normal splicing and decrease the expression (PS3) |
| Segregation data | NA |
| De novo data | NA |
| Allelic data | NA |
| Other database | NA |
| Other data | NA |
| Conclusion | Pathogenic (≥PVS1 and 1 PS) |

NA, not available

The rules for combining criteria to classify sequence variants are from the standards and guidelines published by the American College of Medical Genetics and Genomics and the Association of Molecular Pathology in 2015
